# Implementing a framework for goal setting in community based stroke rehabilitation: a process evaluation

**DOI:** 10.1186/1472-6963-13-190

**Published:** 2013-05-24

**Authors:** Lesley Scobbie, Donald McLean, Diane Dixon, Edward Duncan, Sally Wyke

**Affiliations:** 1 NMAHP Research Unit, Iris Murdoch Building, University of Stirling, Scotland FK9 4LA, UK; 2 ReACH Team, NHS Forth Valley, Scotland, UK; 3 Department of Psychology, University of Strathclyde, Scotland, UK; 4 Institute of Health and Wellbeing, University of Glasgow, Scotland, UK

**Keywords:** Stroke rehabilitation, Goal setting, Process evaluation, Multi-disciplinary team

## Abstract

**Background:**

Goal setting is considered ‘best practice’ in stroke rehabilitation; however, there is no consensus regarding the key components of goal setting interventions or how they should be optimally delivered in practice. We developed a theory-based goal setting and action planning framework (G-AP) to guide goal setting practice. G-AP has 4 stages: goal negotiation, goal setting, action planning & coping planning and appraisal & feedback. All stages are recorded in a patient-held record. In this study we examined the implementation, acceptability and perceived benefits of G-AP in one community rehabilitation team with people recovering from stroke.

**Methods:**

G-AP was implemented for 6 months with 23 stroke patients. In-depth interviews with 8 patients and 8 health professionals were analysed thematically to investigate views of its implementation, acceptability and perceived benefits. Case notes of interviewed patients were analysed descriptively to assess the fidelity of G-AP implementation.

**Results:**

G-AP was mostly implemented according to protocol with deviations noted at the planning and appraisal and feedback stages. Each stage was felt to make a useful contribution to the overall process; however, in practice, goal negotiation and goal setting merged into one stage and the appraisal and feedback stage included an explicit decision making component. Only two issues were raised regarding G-APs acceptability: (i) health professionals were concerned about the impact of goal non-attainment on patient’s well-being (patients did not share their concerns), and (ii) some patients and health professionals found the patient-held record unhelpful. G-AP was felt to have a positive impact on patient goal attainment and professional goal setting practice. Collaborative partnerships between health professionals and patients were apparent throughout the process.

**Conclusions:**

G-AP has been perceived as both beneficial and broadly acceptable in one community rehabilitation team; however, implementation of novel aspects of the framework was inconsistent. The regulatory function of goal non-attainment and the importance of creating flexible partnerships with patients have been highlighted. Further development of the G-AP framework, training package and patient held record is required to address the specific issues highlighted by this process evaluation. Further evaluation of G-AP is required across diverse community rehabilitation settings.

## Background

Goal setting is seen as an essential component of effective and efficient stroke rehabilitation [1,2] and is implemented routinely in practice [3]. As well as creating an ideal opportunity for person-centred care [4], it can increase patient adherence to therapy programmes and optimise goal related behaviour [5-7] Patients with increased involvement in goal setting report greater satisfaction with their rehabilitation experience and that set goals have more personal relevance [8].

Approaches to goal setting have been described within rehabilitation [8-10] and self-management interventions [11,12]. Practice recommendations have been developed to guide writing Specific, Measurable, Achievable, Realistic/Relevant and Timed (SMART) goals [13] and ways to optimise involvement of people with communication difficulties in the process described [14,15]. Outcome measures based on patients’ goals are well known in the clinical and academic arena [16,17].

Despite the prevalence of goal setting in practice there has, until recently, been a distinct lack of theory and evidence base to support its use. Theory and evidence-based approaches to goal setting are now however beginning to emerge. One such development is ‘Good Goals’, which is primarily aimed at improving the access and equity of occupational therapists’ case load management and has so far been developed and tested in paediatric settings [18]. Another is the Goal Setting and Action Planning (G-AP) Framework, which has been developed using a theory-practice based approach within community based stroke rehabilitation [19,20]. The G-AP framework is designed to guide health professionals through a systematic goal setting process with a primary aim of optimising goal attainment and patient involvement.

### Development of the G-AP framework

Development of G-AP has been guided by the Medical Research Council (MRC) Framework for the development and evaluation of complex interventions [21]. The methods used to develop G-AP have been fully presented elsewhere. In summary, they included: (i) a systematic review of the literature to identify psychological constructs with most potential to inform goal setting practice [19], (ii) a causal modelling exercise [22,23] to map these constructs onto a goal setting process [20] and (iii) convening of a multi-disciplinary task group to develop the theoretical goal setting process into a practice framework suitable for use in clinical practice [20]. This included development of a G-AP implementation guide (see Additional file 1) and a patient held record to record each stage of the G-AP process (see Additional files 2, Additional file 3, Additional file 4 and Additional file 5).

Our causal modelling exercise identified four distinct stages of the G-AP framework: goal negotiation, goal setting, action planning & coping planning and appraisal & feedback (see Additional file 6). In the *goal negotiation stage*, patients consider their current situation and identify the main problem(s) they want to address. In the *goal setting stage*, the identified problem is refined into a specific, challenging rehabilitation goal agreed by both health professional and patient. A*ction plans* detail what the patient has to do (in sequential steps) to meet the goal and *coping plans* detail strategies to be activated if barriers hinder action plan attainment. A self-report measure of self-efficacy is included in the planning stage to assess patients’ confidence to complete set plans [11] (pg22); a lack of confidence (score less than 7) suggesting the plan should be modified to optimise the chances of the patient following through with it. Finally, the *appraisal* and *feedback stage* prompts a progress review and feedback from the health professional to the patient. The causal modelling exercise hypothesised that the G-AP framework would optimise patients’ attainment of rehabilitation goals through their successful completion of action plans which would result in incremental improvements in goal sub-skills and self-efficacy [20].

### Implementation of G-AP

Having developed the G-AP framework in a theoretically sound and clinically grounded way, it is now essential to evaluate its implementation within routine clinical practice [21]. Evaluating the implementation of G-AP will enable the systematic identification of any problems associated with its use which can then be addressed prior to further evaluation. We completed a process evaluation of G-AP’s implementation within a community rehabilitation team (ReACH team) in Scotland. Specifically, we aimed to investigate G-AP’s implementation with people recovering from stroke, its acceptability to patients and health professionals and their views about its benefits (if any). Furthermore, we aimed to explore the experience of implementation, identifying the actual practices and interactions that took place within the clinical setting.

## Methods

### Study design

The G-AP framework was implemented by the ReACH team for a 6 month period (Jan-June 2008) with all new stroke patients who would normally be involved in goal setting. Prior to implementation, all team members participated in G-AP training. This consisted of two, one hour sessions which covered use of the G-AP framework, the implementation guide and patient held record. This training was in addition to monthly updates team members received over a ten month period on the stage by stage development of G-AP and patient held record (ReACH Team involvement in G-AP development is described elsewhere [20]).

Following the 6 month implementation period, a cross-sectional process evaluation of the G-AP framework was conducted using mixed methods. Qualitative interviews with stroke patients and health professionals were used to gather insights about their experience of G-AP implementation and uncover their views about its acceptability and impact (if any) on outcomes that were important to them. The case notes of interviewed patients were reviewed to assess the fidelity of G-AP implementation. Health professionals did not know which patients would be interviewed or have their case notes reviewed during the implementation period. Ethical approval was obtained from the University of Stirling ethics committee. This study did not require NHS ethical approval as it was deemed a ‘service evaluation’ of an intervention recognised as current care. All patients provided informed written consent for the interview and case note review; all health professionals provided informed written consent for the interview. The interviews were conducted by a practising member of the clinical team (DMcL) who had secured protected time within his NHS post for this purpose. Patients seen by DMcL were not included in the study in order to minimise response bias.

### The context - ReACH team and ‘usual’ goal setting practice

At the time of this study, the ReACH team had 16 health professionals delivering community rehabilitation services to patients (mostly under the age of 65) in NHS Forth Valley. Four rehabilitation assistants worked alongside health professionals to implement individual rehabilitation programmes. The majority of referrals to the team were for people with neurological problems including stroke, multiple sclerosis and head injury. The length of rehabilitation input was open ended, depending on how much time health professionals judged they need to meet set rehabilitation goals. This could vary from a few weeks to many months and would typically involve two or three visits from the team per week. Approximately six stroke referrals were accepted by the team each month. Only patients requiring multi-disciplinary input were seen by the team – those patients requiring uni-disciplinary input (approximately two referrals per month) were forwarded to other services.

Goal setting was already well-established and highly valued in the ReACH team. Prior to G-AP implementation, the team set goals collaboratively with patients then worked towards them over a period of time. How the process unfolded after the goal setting stage was variable and reflected individual health professional’s preferences. Goal appraisal and feedback was formally implemented at the end of team input when discharge was discussed with the patient. Goal setting information was kept in department based record - patients did not receive a copy of their personal goals. Health professionals discussed patient’s goals in regular department based goal review meetings.

### Participants

#### Patients

All stroke patients seen by the team were eligible for recruitment except for those being treated by DMcL. We expected the experience of G-AP to vary by gender and level of disability so planned to: i) purposively sample 12 patients with equal numbers of males and females; ii) include patients with a range of disability scores (assessed using each patient’s initial Therapy Outcome Measure score [24]); and iii) to include at least two patients with aphasia.

#### Health professionals

All 16 health professionals (five occupational therapists; four physiotherapists; two speech and language therapists; two psychologists; two nurses and one dietician) working in the ReACH team were eligible for recruitment. We aimed to recruit eight of them, representing each professional group as follows: two occupational therapists, two physiotherapists and one each of the other four professions.

### Data collection

Patients and health professional interviews were conducted following the implementation period. Patients were interviewed in their own homes; health professionals were interviewed in a ReACH team interview room. With permission, all interviews were tape-recorded and transcribed verbatim. If necessary, we planned to interview patients with communication impairment using Talking Mats™ (http://www.talkingmats.com), an evidence-based low-tech communication framework routinely used within team.

The interview guides for patients and health professionals were similar (Additional file 7 and Additional file 8). They covered views about participants’ experience of using G-AP, any problems they had using it and perceived benefits or negative consequences of G-AP. All participants were asked to ground their answers in particular examples of goals that they had worked on. This was expected to produce illustrated examples of the practical use of G-AP in relation to real practice rather than at a general level.

Two of the researchers (D McL, LS) conducted the case note review of the interviewed patients. Information relevant to each stage of the G-AP framework was extracted using a data matrix (see Additional file 9). Patients were identified by number only.

### Data analysis

Data was analysed to assess the implementation of G-AP, its acceptability and perceived benefits.

#### *Patients records data*

Data were analysed descriptively in relation to whether there was evidence of implementation of each of the G-AP stages or not.

#### *Interview data*

Interview data were analysed using the Framework approach to thematic analysis [25]. This allowed for the identification of both novel and expected issues within the broad themes of implementation, acceptability and perceived benefits, and facilitated comparison between health professionals and patients. The transcripts of both health professional and patient interviews were anonymised by an administrator not involved in the study. Following anonymisation, one of the research team (LS) listened to each recording to familiarise herself with the data, and checked transcripts for accuracy. LS developed an initial coding framework which was independently applied to 40% (6 /16) of the transcripts by two authors (D McL, DD) and a clinical research colleague not involved in the study (SB). The coding framework was refined following discussion of data within and between codes. Similar codes were grouped and redundant codes removed. The revised coding framework included three broad themes based on the specific research questions (views of the implementation, acceptability and perceived benefits) which allowed the identification of both expected (such as views on the G-AP stages) and unexpected (such as family/ carer input in the process and differences in partnership working) sub-themes.

LS applied the revised coding framework to all transcripts. Data under each main and sub-themes were grouped into a matrix and summarised to ensure the range of views expressed by both patients and health professionals were covered. Unexpected cases or those that did not fit the emerging analysis were examined to seek to refine or refute developing summaries and ensure their credibility.

## Results

### Participant characteristics

Thirty four stroke referrals were accepted by the ReACH team within the study period. Of these, four patients did not require on-going rehabilitation input, six patients required short interventions that were not underpinned by goal setting and one patient refused team input. The G-AP framework was implemented with the remaining 23 patients of which 15 were invited to participate in the study (eight were excluded as they were either being treated by DMcL (n = 6) or were not medically stable (n = 2). Eight patients provided informed consent to participate in the interview and have their case notes reviewed (see Table 1: Patients included in the study). The remaining seven chose not to participate.

**Table 1:** Patients included in the study

**Patient**	**Sex**	**Age**	**Ethnicity**	**Employment pre-CVA**	**Social situation**	Disability level*	**Speech difficulty**	**HPs involved**
**1**	M	64	White Scottish	Unemployed	Lives alone	moderate	yes	PT, OT, SALT
**2**	F	59	White Scottish	Bank clerk	Lives with husband	moderate	no	PT, OT
**3**	M	53	White Scottish	Engineer	Lives with wife	moderate/ severe	yes	SALT, OT, N, D
**4**	M	78	White Scottish	Retired	Lives with wife	moderate	yes	OT, SALT
**5**	F	43	White Scottish	Clerical worker	Lives with husband	moderate	yes	SALT, OT, PT
**6**	M	65	White Scottish	Retired	Lives with wife	moderate	no	PT, OT
**7**	M	56	White Scottish	Driver	Lives alone	mild	yes	SALT, OT, PT
**8**	F	29	White Scottish	Nursing auxiliary	Lives with husband	mild	yes	SALT, OT, PT

*PT* Physiotherapist, *OT* Occupational Therapist, *SALT* Speech and Language Therapist, *N* Nurse, *D* Dietician, *HP* Health Professional. * Based on averaging Therapy Outcome Measure scores across Impairment, Activity, Participation, Wellbeing.

Eight health professionals were invited to participate - two occupational therapists; two physiotherapists, one dietician, one nurse and two speech and language therapists. All agreed and provided informed consent to participate in the interview.

### Implementation and acceptability of G-AP in clinical practice

#### *Fidelity of G-AP implementation*

The case note review suggested that goal negotiation, goal setting and action planning were implemented as intended with all eight patients; however, two aspects of planning - coping planning and measuring confidence to complete action plans - were inconsistently recorded suggesting they were not routinely implemented. Only two of the eight case notes documented use of coping plans. Four of the eight case notes documented measuring confidence to complete plans; however, this was inconsistent and appeared to be done informally rather than using the visual analogue scale. The appraisal and feedback stage was mostly implemented as intended, however inconsistencies were noted. One of the eight case notes did not document an appraisal/ feedback stage in relation to any action plans or goals.

### Practical experience of the G-AP stages

Patient and heath professional views suggested that each stage of the G-AP framework had a distinct purpose and made a useful contribution to the overall process.

#### *Goal negotiation and goal setting*

Although the goal negotiation and goal setting stage had a distinct purpose, they often unfolded as a continual process in practice with problems identified in the former informing specific goals set in the later. For example, Patient 5 talked about how forgetting household chores (for example, ironing her son’s shirt for work) led to a goal about using specific memory strategies to remember daily tasks. Health professional 5 described how a discussion with one patient about her frustration at people completing her sentences for her led to a goal about being able to finish sentences in day to day conversation.

Health professionals said they found the process of identifying *general* problem areas and goals in the goal negotiation stage relatively straight forward, but refining these into a *specific* problems and goals in the goal setting stage was more challenging and influenced by factors such as the patient’s recovery expectations and their cognitive and communication status.

Health professionals described a variety of tools and strategies to facilitate the process of negotiating and setting goals. Of particular importance was the use of Talking Mats® with people who had aphasia as one health professional explained:

***Health professional 2****“M’s got severe communication problems, both receptive and expressive, so just sitting talking to him we would’ve got nowhere … So we used the ‘mats’* [Talking Mats®] *quite early on and got some idea of the areas that he was particularly concerned about and then tried to use it in conjunction with this, the G-AP framework. He could certainly identify what mattered to him using symbols.”*

Other useful tools at this stage included the work sheet entitled “Coming up with the goals” included in the G-AP record (see Additional file 3) and using a blank sheet of paper to develop a visual representation of goal priority areas. Patients and health professionals also reported useful questions or ‘stock phrases’, for example: *“Think about what you would like to be able to achieve by …….* (Future date)*”* (Health Professional 2) or *“What sort of things did you enjoy prior to having the stroke?”* (Patient 4, Patient 8) or *“Think of something very specific to do with that activity* (e.g. cooking) *you would like to work on”* (Health Professional 2). Giving patients examples of potential goals to consider was also seen as useful.

#### *Action planning*

Patients and health professionals described action plans as a series of ‘stepping stones’ or ‘targets’ that created a manageable route to achieving specific goals. For example;

***Patient 2****“A.* [rehabilitation assistant] *used to take me down to* [name of a shop] *and then she’d come round with me, and then she’d take me down, and then she’d stand and watch me, then she’d take me down* [pause 3 secs] *and, and sit in the car, and let me come back. And then I got a taxi and met A. And then the last time I went down and came back in a taxi* [myself].”

Action plans were often viewed as ‘homework’ by patients. Typically, they would be completed by the patient independently (for example, practicing a peg board activity to work on finger dexterity – Patient 3) or with support (for example, supervised practice using the bus – Patient 1). Health professionals reported numerous instances where progress depended on *them* completing an action plan rather than the patient for example, arranging a prescription for a supplement to improve nutritional state (Heath professional 4). Patient and health profession reports suggested patient adherence to action plans was usually high, with some exceptions.

#### *Coping Planning and measuring confidence to complete plans*

For all health professionals, these two aspects of the framework were a new and unfamiliar addition to their clinical practice. Those health professionals who discussed coping plans (only two of the eight health professionals interviewed) viewed them as useful. For example:

***Health professional 7****“I have spoken to folk about barriers. (Em), not every time, but I think it is definitely a useful thing to do. If people think through what might get in their way of them achieving these steps* [action plans], *if they've particular tasks to do, I think (em), you can kind of problem solve if there is a particular barrier.”*

Health professional reports suggested that barriers were sometimes considered in a general way rather than in relation to specific action plans. For example, Staff member 8 explained how she had considered the impact of osteoporosis on her patient’s general ability to achieve rehabilitation goals.

Patients did not refer to coping plans per se, but did discuss strategies they had used to overcome anticipated barriers to successful action plan completion. For example, Patient 4 described how he had identified memory issues as a potential barrier to goal completion: He thought he may forget the steps required to access his on-line banking system. In response to this, the health professional developed a coping plan - she wrote down instructions to access the online banking and encouraged him to use the instructions if he got ‘stuck’ whilst trying to complete his action plan.

Health professionals viewed confidence as an important factor that would influence action plan completion; however, many reported they had not got into the ‘habit’ of using the visual analogue scale or preferred to measure confidence in an informal way as reflected in the following excerpt;

• ***Interviewer:****“Do you use the confidence scale?”*

• ***Health professional 4****: “I don’t”*

• ***Interviewer****: “You don’t?”*

• ***Health professional 4****: “Bad habit - Not having got into the habit of using it. It’s almost doing it without actually formalising it. So I don’t formalise it in terms of giving the individual* [the patient] *a score or asking them how they would score themselves, but I do do it.”*

Some found measuring confidence it a time consuming step at the end of the planning stage. One health professional reported she did not fully understand the purpose of the scale and so was not inclined to use it.

#### *Appraisal and feedback*

Both health professionals and patients viewed this stage as an opportunity to gauge progress; however, some health professional reports suggested that it was implemented intermittently to review goal progress rather than on an action plan by action plan basis.

Patients who judged they were doing well were encouraged. One patient described how she felt after successfully climbing up a step: “*Wow, my leg is not as bad as I thought it was!*” (Patient 2). Conversely, negative self-appraisal was discouraging as highlighted when another patient described how he felt after not achieving his goal of completing a crossword, “*I was just becoming really angry with myself and frustrated.*” (Patient 8).

The feedback health professionals gave to patients was reported to serve a variety of purposes, the main one being to enhance confidence (self-efficacy) through praising success. As one patient explained, *“Every move I made, she said well done, and indeed things cheer you up, it’s amazing what it does psychologically just to say well done!”* (Patient 2). Feedback also provided patients’ with reassurance, for example - *“You’ll get there, don’t worry about it”* (Patient 7) and advice (often about pacing), for example *“You’re giving yourself too much to do, just take your time, take it on a week to week basis and you’ll get there”* (Patient 3). Health professionals reported that the feasibility of implementing the appraisal and feedback stage could be compromised by time constraints.

An important acceptability issue raised by health professionals was that the appraisal and feedback stage made it *explicit* to patients if they were not making progress, and that this could have a negative impact on their well-being. Different strategies used to manage this were reported including, avoiding or not explicitly addressing goals that had not been achieved, re-framing failure in a positive way or providing support and reassurance. For example:

***Health professional 3****“I think you have to be careful about how you deal with that* [goal non-attainment] *with the patient and how you approach it, that you do it in a positive way saying, ‘well OK, this is what we started, this is what we thought, you know, it’s not quite worked out like that, but we’ll go back and we’ll try something else’.”*

Conversely, none of the patients voiced concerns about goal non-attainment or how it might impact on their well-being. Although failure to achieve action plans and goals was said to be disappointing, some patients said they used what they had learned from their experience to re-assess their situation and to consider more realistic goals. For example, one patient worked as a driver and said that getting his driving licence was an important goal for him so he could return to work. However, failing his driving assessment was an important experience that led him to conclude that getting back to work was not a realistic goal.

**Patient 7***“After I had my, my driving assessment, I knew that the information* [information as he was driving the car for example signs and oncoming vehicles] *just wasn’t coming quick enough…. I thought it was doable, but I’ve been realising* [since] *I got through the assessment, and how I done, that I said - this is not going to be doable.”*

#### *Decision making*

Health professional and patient accounts suggested that appraisal and feedback lead to explicit decisions being made about what to do next the basis of whether satisfactory progress was being made or not. Collectively, appraisal, feedback and decision making performed a regulatory or adjusting function within G-AP. If progress was satisfactory - subsequent action plans were set and/or new goal(s) negotiated as illustrated in this health professionals account of a conversation she had with a patient after a successful visit to the local shop:

***Health Professionals 5****“Right, we’ve been to the shop and everything’s gone fine, next time I’m going to get you to walk in [the shop] and I’m going to wait at the door. Are you happy with that?”*

If progress was not satisfactory, new re-targeted plans were set or the goal was downgraded or abandoned. When discussing his lack of progress due to deteriorating health, one patient reflected:

***Patient 1****“We* [the patient and the health professional] *sat down and we discussed it all, you know, but the goals have come down* [been downgraded] *now, know what I mean? It’s just not going to happen, what we thought at first* [going into town on the bus]*.”*

Factors that contributed to goal non-attainment included an unexpected deterioration in physical heath, lack of anticipated recovery from stroke related impairments or underestimating the impact impairments would have on achieving a particular goal.

### The G-AP patient held record

On the whole, patients and health professionals valued the G-AP patient held record. Most patients’ referred to it and said it was particularly important at the beginning of the rehabilitation input where it guided what they practised and helped them monitor progress. Patient 2 referred to the record as her “*bible*” as she looked at it daily to keep her on track with the action plans she had to work on – even taking it on holiday for reference. Some also suggested that the G-AP record allowed family members to find out about the goals and action plans in place, creating an opportunity for them to consider how they could contribute to the process, for example, suggesting new action plans and /or encouraging and supporting their family member to complete them. Those patients who did not use the G-AP record said they preferred to discuss goals and action plans with the health professional and commit them to memory rather than paper. A marginal but important view expressed by one patient with cognitive difficulties was that the G-AP record was confusing and an annoyance;

***Patient 8 “****I just feel, feel there is so much paperwork here, here and I get confused with it and tha, that’s me, I’m not a novice to pay, paperwork believe you me, but I feel that there’s just too much there and my, my some, some, sometimes my concentration levels are poor and to sit, I’ve got to sit and really think,* (er), *right* [going through papers] *no wait a minute look for* [goal] *two.”*

Health professionals also reported benefits of using the patient held record, for example:

***Health professional 5****“I think the folder’s* [G-AP patient held record] *a great idea … I have always felt very strongly that the people that we work with should have something to refer to.... And they need to have notes of what our expectations are of them so it works extremely well from that point of view. It’s something that they each focus on when we meet at review and whatever, and see the progress they’re making.”*

It was seen to prompt implementation of each stage of the G-AP framework and to enhance interdisciplinary working (for example, setting goals in the context of those already set or suggesting action plans under goals set by other professionals). However, some health professionals reported a logistical problem getting information written in the G-AP record back into patients’ department based service records (in spite of using carbon paper sheets within the record). This negatively impacted on team discussion at weekly goal review meetings which was viewed as a significant problem. They also noted that patients with reading difficulties did not benefit from a written record of their goals and action plans. Finally, some health professionals reported that writing goals and action plans in the G-AP record was a new and added task for them which required extra time to complete and had not been habitually integrated into their routine practice.

### Views on factors that facilitated and inhibited G-AP implementation

A sub-theme identified from the data was the factors that facilitated and inhibited use of G-AP in clinical practice. These could be grouped under the headings of health professional factors, patient factors and process factors (summarised in Table 2: Factors that facilitated/ hindered use of the G-AP framework). Facilitating factors included: patients having previous experience of goal setting, health professionals being confident in their goal setting abilities and rehabilitation assistant involvement in the process. Inhibitory factors included: patients who felt emotionally overwhelmed with the consequences of stroke, health professionals lacking experience in post stroke recovery and work-load pressures. A particular inhibitory factor identified by health professionals was severe receptive and expressive aphasia. In these instances, professionals said they tended to use G-AP with family members on the patients behalf. All of these factors interacted to create an optimal or less than optimal condition for G-AP implementation.

**Table 2:** Factors that facilitated/ hindered use of the G-AP framework

	**Facilitators**	**Inhibitors**
**Patient factors**	• Previous experience goal setting	• Cognitive impairment e.g. poor insight, executive dysfunction
	• Familiarity with the G-AP process	• Communication difficulties
	• Being in the ‘right frame of mind’	• Complex emotional/ social/ health issues
		• Unrealistic expectations
**Process factors**	• Rehabilitation assistant involvement	• Individual health professionals’ waiting lists resulting in team members initiating input at different times
	• Goal meetings in the patent’s house	• Time pressures leading to incomplete implementation of the process
	• Consistent use of G-AP record	• Staff absence
	• Explaining the G-AP process to patients at the outset	
**HP factors**	• Experience of using goal setting	• Lack of experience using goal setting
	• Experience of post stroke recovery	• Lack of experience of post stroke recovery
	• Confidence in goal setting abilities	• Not habitually using G-AP in routine practice
		• Lack of confidence using G-AP
**Other**		• HP and patient having differing views about priorities and/or what constitutes improvement

*HP* Health Professional.

### Partnership working

A second sub-theme within the health professional and patient accounts was the bespoke and dynamic nature of partnerships between health professionals and patients. Respondents talked about differing roles in the partnership. Patients described their main role as informing health professionals about their goal priorities and giving them feedback about what they felt they could and couldn’t achieve. Health professionals described their main role as guiding and encouraging patients through the G-AP stages, for example helping them to tailor unrealistic or general goals into specific, achievable goals and providing education and information that would help them make informed goal choices.

Accounts from patients and health professionals also suggested that a continuum existed in relation to who took the *lead* during the G-AP process with ‘patient led’ at one end and ‘health professional led’ at the other. When patients preferred health professionals to take the lead, they said that health professionals were the ‘experts’ with experience of dealing with other people in the same situation, or had specialist knowledge that made them better placed to suggest goals that would help them in their recovery. When asked about setting goals, Patient 2 said: *“I went along with E* (physiotherapist); *she was right 100% like you know.”* When patients took the lead, they tended to have experience of setting goals, either in a previous life context (for example, in a previous job or hobby) or during their current rehabilitation episode. They also had clear ideas about valued activities they wanted to resume and a belief that recovery, to a large extent, was dependent on their own efforts as Patient 2 explained, *“It’s in here, in my head really, my own attitude has got to be right to get myself where I want to be”.* Regardless of who led, both groups described each stage of the process as collaborative with agreed goals and action plans reflecting patients’ priorities and unique personal circumstances.

### Perceived benefits of G-AP

Patients primarily judged the effectiveness of G-AP on the basis of whether they were able to carry out their goals as planned. When asked to explain how she knew that G-AP had worked for her, Patient 5 said in relation to her goal of returning to getting her shopping at the supermarket: *“Because I was doing it, and pleased to be doing it.*” Patients described how identifying personal goals and action plans increased their motivation by acting as an incentive – something to aim for. A repeated view was that achieving goals and action plans produced a sense of achievement and an important boost in confidence. For example;

• **Patient 8**: *“When, when you manage to achieve that goal you think, oh yes well I can go, go, go a wee bit further now.”*

• **Interviewer***: “And was that positive?”*

• **Patient 8**: *“yeah, yup because right at the beginning of the process you feel so neg, neg, negative and you feel how am I going to get my life ba, back together again?”*

A general view held by patients was that the positive relationship they had established with health professionals was a significant factor that contributed to their recovery.

Health professionals talked about the benefits of G-AP at the patient and practice level. There was a prevalent view that the collaborative nature of the G-AP process helped patients have a greater sense of control and participation in their rehabilitation. Additionally, it was felt that patients were more focused on their goals, which had a positive impact on their motivation and adherence to the goal plan. Health professionals perceived their practice to be more patient centred (with goals set reflecting patient rather than professional priorities), goal focused and efficient (due timely changes being made to the goal plan if progress was not being made).

## Discussion

The results of this process evaluation provide preliminary support for the clinical usefulness of G-AP. It is broadly acceptable and has perceived benefits from both patient and health professional perspectives. However, the evaluation highlighted areas in which G-AP could be improved. We describe how we have addressed each area for improvement below and discuss our findings in relation to partnership working when using the G-AP framework.

### Combining goal negotiation & goal setting and making decision making explicit

**Figure 1:**
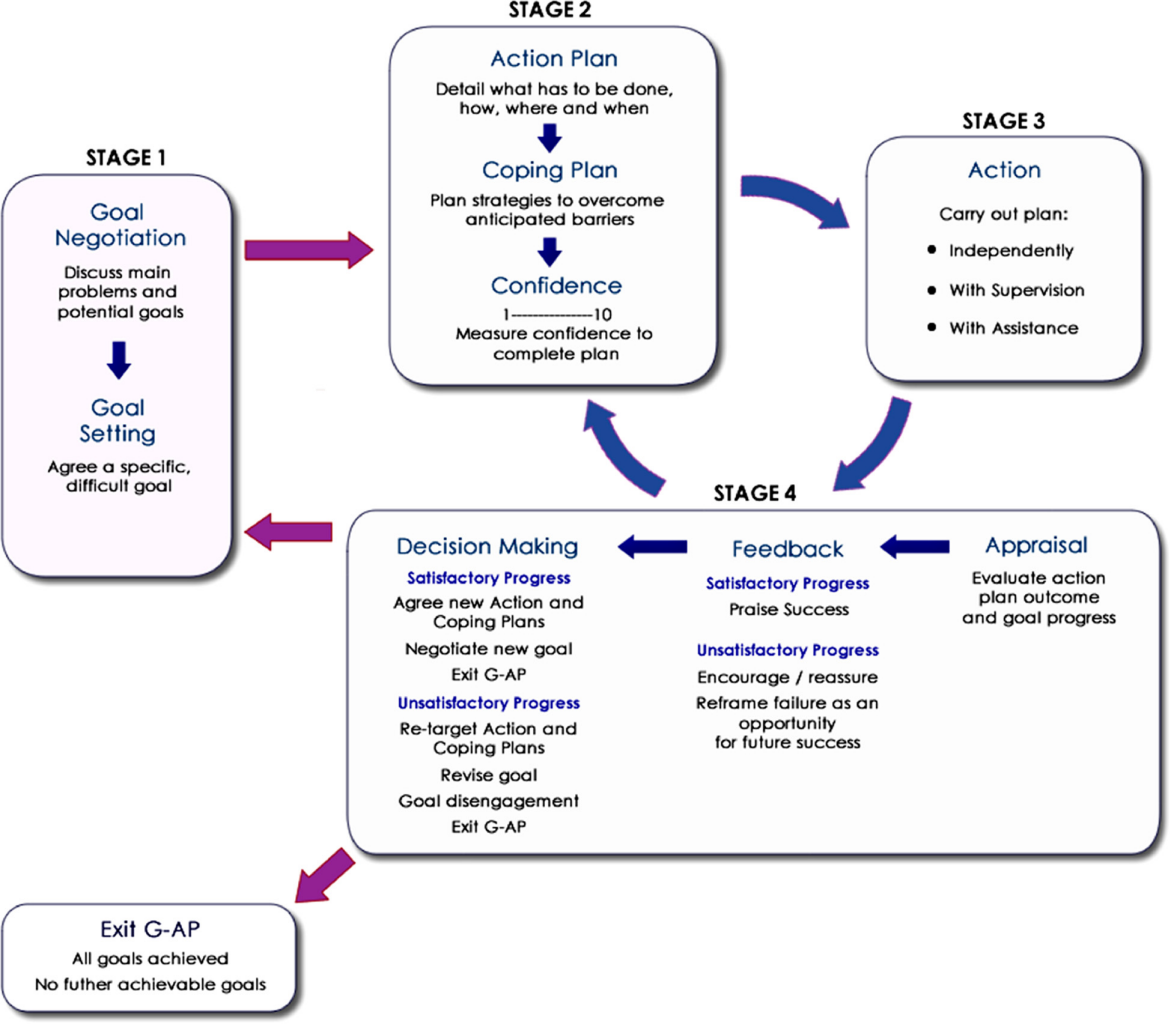
**Revised G-AP framework.** The revised illustration of the G-AP framework merges goal negotiation and goal setting into one stage and includes an explicit decision making component in the appraisal and feedback stage.

To optimise its usefulness to health professionals, we have revised the visual illustration of the G-AP framework to better reflect how the process unfolds in practice. Goal negotiation and goal setting, whilst remaining distinct components of the process, have been merged into one stage and an explicit decision making component included in the appraisal and feedback stage to clarify options available when progress is judged to be either satisfactory or not (see Figure 1 – The revised G-AP framework).

### The experience of goal non-attainment

Our results highlighted health professionals’ concerns about the impact of goal non-attainment on patients’ emotional well-being. We believe that failure to achieve goals is inevitable in stroke rehabilitation, because neither patients nor health professionals can foresee some of the factors that may render goals unachievable or predict with absolute accuracy what goals can be achieved at some future point.

The tension that health professionals have to manage when trying to maintain patients’ hope and motivation whilst at the same time dealing with disappointment and fostering realistic expectations about the future has been highlighted [26,27] This is indeed a difficult balancing act that has to be managed on a patient by patient basis. Our patient data suggested that failure to achieve set goals *did* lead to disappointment and frustration; however, this experience helped them to understand and accept their limitations and disengage from un-attainable goals. These findings raise the possibility that, for some patients, goal non-attainment may be a valuable and necessary part of the rehabilitation process.

This is consistent with the Social Cognitive Theory perspective which views satisfaction of goal accomplishments and dissatisfaction of failure as important outcomes that will influence a person’s motivation to act in new ways to increases the likelihood of future goal success [28]. An improved G-AP training programme will highlight the regulatory function of goal non-attainment.

### Optimising implementation of G-AP stages

The goal negotiation, goal setting and action planning stages of the framework were routinely implemented. This is perhaps not surprising as these aspects of the goal setting process are well documented and established in practice, albeit action plans often being referred to as short term goals within this literature [26,29-32]. This evaluation showed that it was the novel additions to practice - coping planning, measuring confidence to complete action plans and appraisal and feedback on an action plan by action plan basis – that were not always implemented. Health professionals reported a number of factors to explain this including not having got into the habit of routinely implementing these aspects of the framework, not fully understanding their purpose or time constraints.

Whilst health professionals are likely to consider the issues of barriers, coping, confidence and feedback in routine practice; use of the G-AP framework requires a targeted, systematic approach. Although the theoretical justification for this approach has been described [20], the G-AP training delivered prior to the implementation period may not have covered these aspects of the framework in enough detail or highlighted the subtle but important differences between routine goal setting practice and that informed by the G-AP framework.

Previous research has highlighted the importance of health professionals acquiring the necessary knowledge, skills and habits for effective implementation of evidence based practice [33]. Improved G-AP training will focus on enhancing health professionals’ knowledge of novel aspects of the framework, how they differ from ‘usual’ practice and why they are important. Improved training will also provide opportunities for skills development by practicing implementation of these specific stages in clinical role play scenarios. To facilitate habitual implementation of novel stages, the G-AP patient held record will be revised to include a visual prompt for health professionals to consider the need for a coping plan, measure patient’s confidence at the action planning stage and to complete appraisal and feedback following action plan completion. Barriers to implementation will be identified within the training and potential solutions explored and developed. Finally, the G-AP implementation protocol will be revised so that measuring confidence to complete action plans can be done formally (using the visual analogue scale) or informally (by just asking patients how confident they feel) depending on the health professionals judgement of which would be the most helpful.

### Use of the G-AP patient held record

Patients do not typically have a record of the rehabilitation goals they are working towards [3]. The G-AP patient held record sought to address this issue within the ReACH team. Although it was generally well received by health professionals and patients, important acceptability issues were raised. The record will be re-designed to resolve the logistical issue of having information documented within the record available for team use (for example, during team based goal review meetings). Additionally, future use of the record will be sensitive to individual patient’s views of its perceived usefulness (particularly those who have cognitive and /or communication difficulties) and will aim to facilitate family member involvement in the process if both parties are agreeable.

### Partnership working when using the G-AP framework

Of particular interest are our findings on partnership working. Previous research has shown that patients’ recovering from stroke want to be actively involved in goal setting [34]. This study has shown that feeling involved can incorporate both patient and professionally-led approaches and that this will vary between patients at different stages of the process, and between different goals. As suggested in relation to shared decision making [35] and decisions about screening [36] health professionals should be flexible in their approach to allow patients to engage in the partnership in a dynamic way, and to lead or be led, depending on their preference at that particular time.

### Limitations of this study

Four main limitations of this study have been identified. Firstly, the set up and operation of community rehabilitation teams in the United Kingdom is highly variable [37] – conducting a process evaluation of G-AP in one setting has been a sensible starting point, but does not demonstrate that G-AP could be successfully implemented in the range of community rehabilitation teams currently providing services to people recovering from stroke, particularly in the over 65 age group which is more representative of the stroke population [2].

Secondly, we have tried to embed development and evaluation of the G-AP framework within the clinical setting; hence our continued work with the ReACH team in NHS Forth Valley. We hope this has resulted in an evaluation that is both robust and clinically focussed; however, we acknowledge that conducting the evaluation within ReACH team and having DMcL conduct health professional interviews introduced the potential for respondent bias. We feel this potential was minimised by the engagement of the ReACH team through-out the development and evaluation process. This fostered a strong commitment within the team to complete an evaluation of the G-AP framework that was both accurate and transparent.

Thirdly, a small number of case notes were reviewed to assess the fidelity of G-AP implementation. In accordance with our consent procedures, the case note review was limited to patients who had consented to be interviewed. A separate consent procedure for this aspect of the study may have resulted in a larger number of patients consenting to their case notes being reviewed thus strengthening our evaluation of the fidelity of G-AP implementation.

Finally, our study sample of patients and health professionals was small, with patients in the over 65 age group being under-represented. Additionally, only two of the eight health professionals commented on coping planning. Consequently, we cannot be certain that we have reached data saturation within all themes or that our findings are equally relevant to those people recovering from stroke in the older age groups.

All of these limitations will be addressed by conducting future evaluation of G-AP: (i) in diverse teams that have had no prior exposure to its development or the researchers conducting the study (ii) with stroke patients over the age of 65 and (iii) with a revised consent procedure for the case note review.

### Implications for clinical practice

The findings of this study support the inclusion of goal negotiation, goal setting, planning and appraisal, feedback & decision making when using the G-AP framework community based stroke rehabilitation. They also highlight the regulatory function of goal non-attainment and the need for health professionals to be confident they can manage both success and failure to achieve goals in clinical practice. Finally, we believe that health professionals should be flexible in their partnerships with patients, and be open to both patient and professionally led approaches.

### Implications for future research

The importance of understanding how complex interventions operate and impact at the patient, health professional and service level has been emphasised [38]. It was reassuring that, as we predicted, the perceived benefits of G-AP reported by patients and health professionals included the positive impact of action plan attainment on self-efficacy and goal attainment. However, our findings highlighted the need to look beyond outcomes at the patient level, and to consider the impact of G-AP at the level of the health professional (for example, more efficient work practices), the family/ and or care giver level (for example, increased participation in the process) and at the team level (for example, improved inter-disciplinary working). These findings will be an important consideration when designing a future study to examine the effectiveness of G-AP in a controlled trial.

## Conclusion

G-AP has been perceived as both beneficial and broadly acceptable in one community rehabilitation team; however, implementation of novel aspects of the framework was inconsistent. The regulatory function of goal non-attainment and the importance of creating flexible partnerships with patients have been highlighted. We have developed the G-AP visual illustration and plan specific revisions to G-AP training and patient held record in response to our findings. We are now in the process of developing an evaluation of the revised G-AP on a larger scale across diverse team settings.

## Abbreviations

SMART: Specific, measurable, achievable, realistic/relevant and timed; G-AP: Goal setting and action planning; MRC: Medical research council; NHS: National health service.

## Competing interests

The authors declare they have no competing interests.

## Authors’ contributions

LS developed study concept, carried out data analysis and interpretation and drafting of manuscript. SW developed study concept and design, assisted in data analysis and interpretation and completed critical revision to paper. DD developed study concept and design, assisted in data analysis and interpretation and completed critical revision to paper. D McL developed study concept and design, completed acquisition of data and assisted in data analysis. ED assisted in interpretation of data and made critical revisions to paper. All authors read and approved the final manuscript.

## Pre-publication history

The pre-publication history for this paper can be accessed here:

http://www.biomedcentral.com/1472-6963/13/190/prepub

## Supplementary Material

Additional file 1 G-AP implementation guide. Click here for file

Additional file 2 G-AP patient held record. Click here for file

Additional file 3 G-AP patient held record. Click here for file

Additional file 4 G-AP patient held record. Click here for file

Additional file 5 G-AP patient held record. Click here for file

Additional file 6 Original G-AP framework. Click here for file

Additional file 7 Patient interview guide. Click here for file

Additional file 8 Professional interview guide. Click here for file

Additional file 9 Data extraction matrix. Click here for file
