# Supplementary figures and images for: Implementing a framework for goal setting in community based stroke rehabilitation: a process evaluation
Source: BMC Health Serv Res. 2013 May 24;13:190. doi: 10.1186/1472-6963-13-190 (PMC3671148; doi:10.1186/1472-6963-13-190)

# Goal setting and action planning process

● G-AP

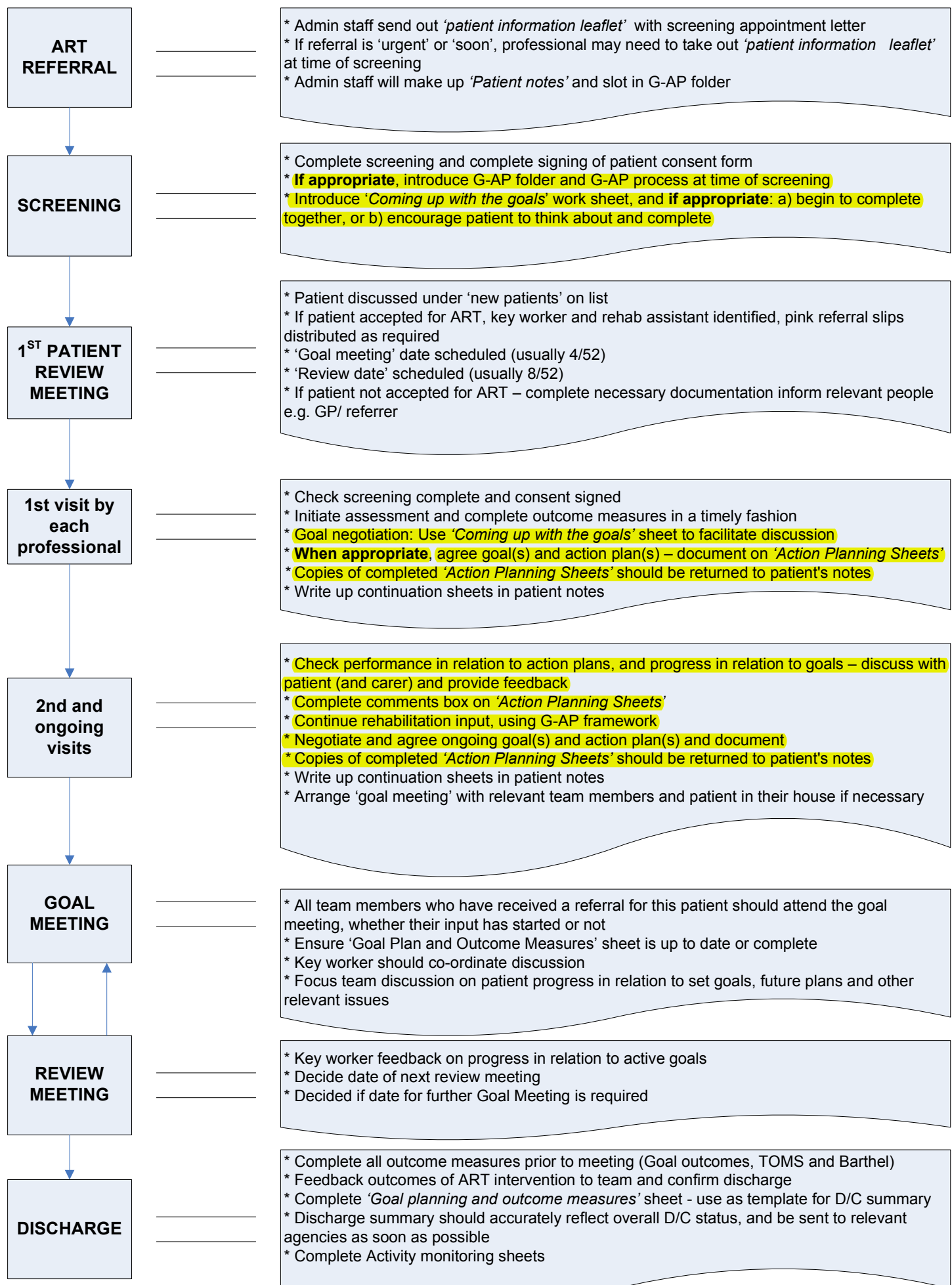

Supplement: Additional file 1 — G-AP implementation guide. [file 1472-6963-13-190-S1.pdf]
