# Supplementary material for: Implementing a framework for goal setting in community based stroke rehabilitation: a process evaluation
Source: BMC Health Serv Res. 2013 May 24;13:190. doi: 10.1186/1472-6963-13-190 (PMC3671148; doi:10.1186/1472-6963-13-190)
Supplement: Additional file 2 — G-AP patient held record. [file 1472-6963-13-190-S2.pdf]

GOAL SETTING…

NHS Forth Valley

What is goal setting?

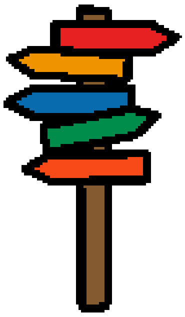

Goal setting involves everyone (that is you, the people who are important to you and the rehabilitation team) working together. We will talk about and agree on the goals you hope to achieve, and changes you would like to make.

What can you do?

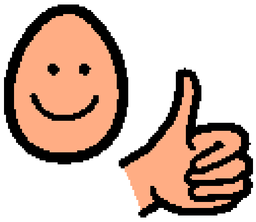

Tell us a bit about yourself and what is important to you. Think about where you're at right now, and where you'd like to get to. If you can, write down your goals in the rehabilitation folder… there are no rights or wrongs!

What will the rehabilitation team do?

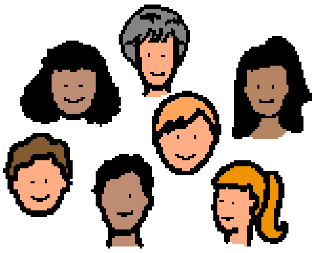

We will find out how you have been doing, and explore the specific difficulties you've been facing. Then we can decide on the most important things that you'd like to work on. To keep us on track, we will agree on the small steps that will guide us along the way.

When the team input will finish

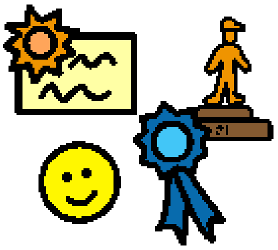

Once you've worked through you're goals and the team input is complete, you will be discharged. If you need advice further down the line, then you can contact us.
