# Supplementary material for: Implementing a framework for goal setting in community based stroke rehabilitation: a process evaluation
Source: BMC Health Serv Res. 2013 May 24;13:190. doi: 10.1186/1472-6963-13-190 (PMC3671148; doi:10.1186/1472-6963-13-190)
Supplement: Additional file 3 — G-AP patient held record. [file 1472-6963-13-190-S3.pdf]

Coming up with the goals….

1. What are your main difficulties?

“This is where I’m at just now”

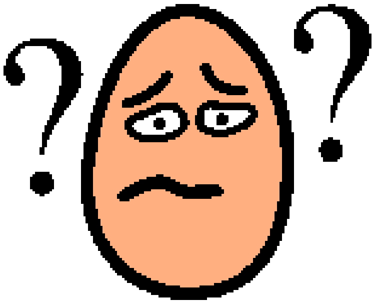

-
-
-
-
-
-

2. What are your specific goals?

“This is where I’d like to get to”

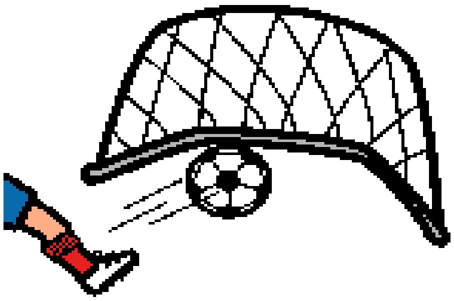

-
-
-
-
-
-
