# Supplementary material for: Implementing a framework for goal setting in community based stroke rehabilitation: a process evaluation
Source: BMC Health Serv Res. 2013 May 24;13:190. doi: 10.1186/1472-6963-13-190 (PMC3671148; doi:10.1186/1472-6963-13-190)
Supplement: Additional file 4 — G-AP patient held record. [file 1472-6963-13-190-S4.pdf]

Area Rehabilitation Team

GOAL and ACTION PLANNING SHEET

NHS Forth Valley

Name: ....

**SPECIFIC GOAL:**<br>*"This is the goal I'd like to achieve"*<br>**Goal Number:**	**OUTCOME**<br>**Date:**
Date set:	Achieved ☐<br>Partially achieved ☐<br>Not Achieved ☐

**ACTION PLAN**<br>*"This is how I'm going to go about achieving it"*	**SUCCESS?**	**COMMENTS**<br>*"How did I get on?"*
Date Set: Target Date:<br>*Confidence level 0 - 10*<br>*Any barriers that might get in your way?*	**YES**<br>**NO**<br>**NOT QUITE**	
Date Set: Target Date:<br>*Confidence level 0 - 10*<br>*Any barriers that might get in your way?*	**YES**<br>**NO**<br>**NOT QUITE**	
Date Set: Target Date:<br>*Confidence level 0 - 10*<br>*Any barriers that might get in your way?*	**YES**<br>**NO**<br>**NOT QUITE**	
