# Supplementary material for: Implementing a framework for goal setting in community based stroke rehabilitation: a process evaluation
Source: BMC Health Serv Res. 2013 May 24;13:190. doi: 10.1186/1472-6963-13-190 (PMC3671148; doi:10.1186/1472-6963-13-190)
Supplement: Additional file 5 — G-AP patient held record. [file 1472-6963-13-190-S5.pdf]

CONFIDENCE SCALE

0 --- 5 --- 10

NOT AT ALL CONFIDENT	SOMEWHAT CONFIDENT	TOTALLY CONFIDENT

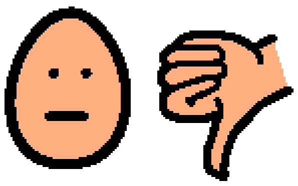

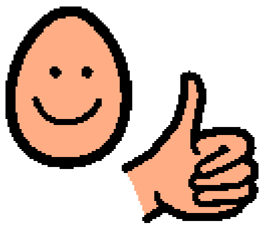

Question: How confident are you that you can……
