# Supplementary material for: Implementing a framework for goal setting in community based stroke rehabilitation: a process evaluation
Source: BMC Health Serv Res. 2013 May 24;13:190. doi: 10.1186/1472-6963-13-190 (PMC3671148; doi:10.1186/1472-6963-13-190)
Supplement: Additional file 6 — Original G-AP framework. [file 1472-6963-13-190-S6.pdf]

Additional File 6: Original G-AP Framework[1]

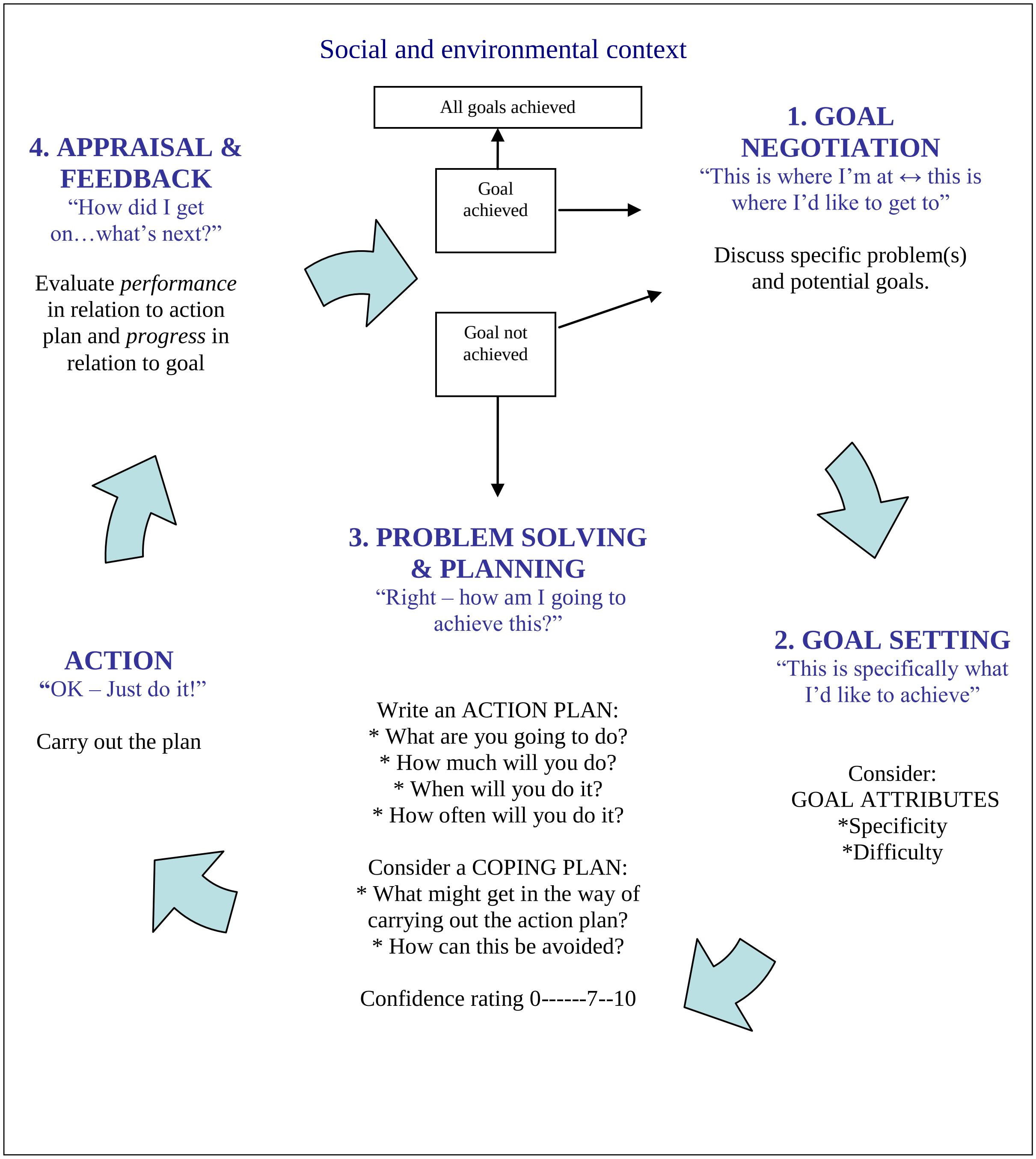

1. Originally published in Clinical Rehabilitation: Scobbie, L., Wyke, S., Dixon, D. Goal setting and action planning in clinical rehabilitation: Development of a theoretically informed practice framework. Clinical Rehabilitation 2011; 25(5) 468–482. Copyright © 2011, SAGE Publications. All rights reserved. http://cre.sagepub.com/content/25/5/468.abstract
