# Supplementary material for: Implementing a framework for goal setting in community based stroke rehabilitation: a process evaluation
Source: BMC Health Serv Res. 2013 May 24;13:190. doi: 10.1186/1472-6963-13-190 (PMC3671148; doi:10.1186/1472-6963-13-190)
Supplement: Additional file 7 — Patient interview guide. [file 1472-6963-13-190-S7.pdf]

Additional File 7.

Patient Interview Schedule: Introduction: You have been involved in goal setting with the Area Rehabilitation Team*. We want to find out what you thought of the goal setting process and if it helped during your rehabilitation.

Areas to be covered include:

1. Experience of G-AP

Patients will be asked to express their views on each stage in the G-AP process and to ground their answers in goals they had worked on. This will include their views on the utility and acceptability of the G-AP process overall and on each component of G-AP, specifically: (Note: Adjust the interview schedule to use language that the patient understand and/or prefer e.g. targets/aims instead of goals.)

a) Goal Negotiation: *Can you talk me through how you identified the areas or problems you wanted to work on?*

b) Goal Setting: *How easy did you find it to come up with goals?*
c) Action Planning and Coping Planning: *How easy did you find it to come up with action plans?; Did your therapist use a scale to find out how confident you were at carrying out your action plan?; Did your plan translate into something you were actually able to do?; Did you think of things that might get in the way of carrying out you action plan?*
d) Appraisal and Feedback: *How easy was it to reflect on what you'd done, and how that tied in with your goal?*

2. Benefits of G-AP

Did the patient experience any benefits associated with their participation in G-AP? If necessary the patient should be asked about the following:
a) The benefits of participating in the G-AP process itself: *Did it help you manage your rehabilitation?*
b) The benefits to their health, in terms of the level of impairment, activity limitations and participation restrictions: *Do you think the G-AP process helped your ability to do the things that are important to you?*
c) What, in their opinion, would improve the benefits they experienced as a result of participating in G-AP: *What, in your opinion, would improve the benefits you experienced from participating in the G-AP process?*

3. Problems associated with G-AP

Did the patient experience any problems associated with their participation in G-AP? If necessary the patient should be asked about the following:
a) Problems associated with their participation in G-AP: *Did you have any problems from using the G-AP?*
b) Any negative consequences on their health, in terms of the level of impairment, activity limitations and participation restrictions?: *Did the taking part in the G-AP process have any negatively effect on you?; Which aspects of G-AP could be changed to reduce or eliminate these problems?*

4. Additional questions

a) How much help or support did you need from the Area Rehab Health professional to go through the whole process?
b) Would you be able to go through the process yourself now, to set your own goals?
c) How many therapists were involved with you?
d) Did they all goal set with you?
e) How useful did you find the patient folder and paperwork?
f) Was it helpful for others working with you from out-with the Area Rehabilitation Team?

* Now known as ReACH Team
