# Supplementary material for: Implementing a framework for goal setting in community based stroke rehabilitation: a process evaluation
Source: BMC Health Serv Res. 2013 May 24;13:190. doi: 10.1186/1472-6963-13-190 (PMC3671148; doi:10.1186/1472-6963-13-190)
Supplement: Additional file 8 — Professional interview guide. [file 1472-6963-13-190-S8.pdf]

**<u>Additional file 8.</u>**

**Health professional interview guide**

***Introduction***

You have been involved in delivering goal setting with the Area Rehabilitation Team with patients recovering from stroke. We want to find out what you think of the Goal Setting process generally and more specifically your experiences with it thinking about specific goals you have worked on with patients. We want to know your opinions on how it works in practice.

1. ***Goal negotiation***
- How easy did you find it to do this with patients (there is quiet a skill to this)?
- Do you think some patients are better at doing it than others? Why?
- How easy was it for patients to establish their problems and where they want to get to?

2. ***Goal setting***
- How easily did the patient establish specific goals?
- Were they realistic in your opinion?
- How much guiding did you need to do?

3. ***Action and coping planning***
- How easy was it for the patients to decide how they were going to achieve their goal?
- How much guiding did you need to do?
- Was it realistic in your opinion?

4. ***Appraisal and feedback***
- How realistic were patients in their achievements (or failures)?
- How successful were they?
- How did they react to success and/ or lack of success when pursuing goals?

5. ***What elements of the G-AP are effective (if any)?***

- In what way do you feel the G-AP works well?

6. ***What elements of the G-AP are ineffective (if any)?***
- In what way do you feel the G-AP doesn't work well?

***7. Other Questions***

- Where does it come in your priorities?
- How much is time an issue?
- What other pressures are on you?
- Do you find the G-AP a pressure?
- Do you think some professionals are better at it? Why?
- Does the G-AP paperwork work?
- How easy is it to capture the on-going goal setting?
- Does the goal setting meeting work?
- Do you think there is a net benefit from doing the G-AP?

**Close the interview; thank the participant for their participation in the project.**
