# Supplementary material for: Implementing a framework for goal setting in community based stroke rehabilitation: a process evaluation
Source: BMC Health Serv Res. 2013 May 24;13:190. doi: 10.1186/1472-6963-13-190 (PMC3671148; doi:10.1186/1472-6963-13-190)
Supplement: Additional file 9 — Data extraction matrix. [file 1472-6963-13-190-S9.pdf]

Additional file 9: Data Extraction Matrix

**Anonymised Patient ID:**			
**Question**	**yes**	**no**	**comments**
Was there evidence of goal negotiation?			
Were specific goals set by the health professionals involved?			
Was a predicted outcome date set in relation to the goal?			
Were action plans set in relation to goals?			
Were coping plans set in relation to action plans?			
Was confidence measured in relation to action plans?			
Was performance appraised and feedback given?			
Were subsequent goals set?			
Any other relevant information?			
